# Shrinkage Stress and Temperature Variation in Resin Composites Cured via Different Photoactivation Methods: Insights for Standardisation of the Photopolymerisation

**DOI:** 10.3390/polym13132065

**Published:** 2021-06-23

**Authors:** Guilherme dos Santos Sousa, Gabriel Felipe Guimarães, Edilmar Marcelino, José Eduardo Petit Rodokas, Arilson José de Oliveira Júnior, Ivana Cesarino, Alcides Lopes Leão, Carla dos Santos Riccardi, Mohammad Arjmand, Rafael Plana Simões

**Affiliations:** 1 Medical School, Sao Paulo State University (UNESP), Avenue Prof. Mário Rubens Guimarães Montenegro, s/n, Botucatu 18618-687, Brazil; guiss13@hotmail.com (G.d.S.S.); edilmar.marcelino@ig.com.br (E.M.); eduardo.rodokas@unesp.br (J.E.P.R.); 2 Department of Bioprocess and Biotechnology, School of Agriculture, Sao Paulo State University (UNESP), Street José Barbosa de Barros, 1780, Botucatu 18610-034, Brazil; gabriel_lhp1204@hotmail.com (G.F.G.); arilsonjr@outlook.com (A.J.d.O.J.); ivana.cesarino@unesp.br (I.C.); alcides.leao@unesp.br (A.L.L.); carla.riccardi@unesp.br (C.d.S.R.); 3 School of Engineering, Sao Paulo State University (UNESP), Avenue Eng. Luiz Edmundo Carrijo Coube, 14-01, Bauru 17033-360, Brazil; 4 School of Engineering, University of British Columbia (UBC), 3333 University Way, Kelowna, BC V1V 1V7, Canada; mohammad.arjmand@ubc.ca

**Keywords:** dental restoration failure, light-curing of dental adhesives, synthetic resins, light-curing of dental resins, photoactivation protocol

## Abstract

The literature has shown that there is no consensus regarding the best resin composite photoactivation protocol. This study evaluated the efficiency of the conventional, soft-start, pulse-delay and exponential protocols for photoactivation of resin composites in reducing the shrinkage stress and temperature variation during the photopolymerisation. The photoactivation processes were performed using a photocuring unit and a smartphone app developed to control the irradiance according each photoactivation protocol. These photoactivation methods were evaluated applying photoactivation energies recommended by the resins manufactures. Three brands of resin composites were analysed: Z-250, Charisma and Ultrafill. The cure effectiveness was evaluated through depth of cure experiments. All results were statistically evaluated using one-way and multi-factor analysis of variance (ANOVA). The use of exponential and pulse-delay methods resulted in a significant reduction of the shrinkage stress for all evaluated resins; however, the pulse-delay method required too long a photoactivation time. The increases on the temperature were lower when the exponential photoactivation was applied; however, the temperature variation for all photoactivation protocols was not enough to cause damage in the restoration area. The evaluation of the depth of cure showed that all photoactivation protocols resulted in cured resins with equivalent hardness, indicating that the choice of an alternative photoactivation protocol did not harm the polymerisation. In this way, the results showed the exponential protocol as the best photoactivation technique for practical applications.

## 1. Introduction

Resins composites are widely used by dentistry professionals for direct dental restorations [1,2]. These resins are composed of monomers that are converted into polymers by a process called polymerisation [3,4,5,6]. The most common polymerisation processes are light-activated processes, and there are different photoactivation techniques [7]. The irradiance emitted by different models/brands of curing devices is variable, usually ranging between 400–1200 mW/cm^2^ [7]. Nowadays, there are devices able to emit light with irradiance up to 1800 mW/cm^2^ [8]. However, high irradiance does not necessarily lead to better curing results obtained by the polymerisation processes [9,10].

Some studies have reported that unsatisfactory behaviour and properties of resin composites, such as excessive polymerisation shrinkage, can be induced by high levels of irradiance during the photoactivation process [7]. The polymerisation shrinkage is the main reason for the low durability of dental restorations made from resin composites [11]. Over time, polymerisation shrinkage can induce microleakages at the tooth and the resin composite interface, which can lead to the recurrence of caries [12]. In addition, the temperature of the resin and in the areas near the material application is increased during the photoactivation process. It is noteworthy that the temperature increase is usually induced by the high levels of irradiance during the photoactivation process [13]. The most-affected areas are teeth and gingiva, with potential risk of damage to the tooth pulp and adjacent tissues [14]. The literature suggests several different photoactivation protocols, with variation of exposure time, distance of application and region of incidence [15,16]. However, there is no standard protocol for photoactivation to minimise these adverse effects; thus, an increase in temperature and polymerisation shrinkage are common occurrences when using LED devices [3,7].

Recent studies have demonstrated that control of irradiance during the polymerisation process can contribute to an improvement in the mechanical properties of the cured biomaterials, and one of the main goals in this research area is to identify an optimal protocol for photoactivation of resins composites [17]. Guimarães, et al. [10] developed an alternative photoactivation protocol named the exponential protocol, in which the LED irradiance along the photoactivation was determined and modelled by a mathematical function (this function being an exponential growth of irradiance in time domain). Their results showed that the proposed photoactivation method minimised the polymerisation shrinkage stress and its effects, without affecting the effective conversion of monomers into polymers. However, the reciprocity law was not considered in their study, and the total activation energies were different for the compared photoactivation methods. Thus, the present study aims to evaluate and compare the patterns of shrinkage stress and, additionally, the temperature variation on resin composites using the most popular photoactivation methods: conventional, soft-start, pulse-delay and exponential. For a better comparison, it was proposed obeying the reciprocity law, adjusting the photoactivation energy and time according to parameters recommended by the composite manufacturers. However, to evaluate new photoactivation protocols, it is also necessary to evaluate cure effectiveness, which is associated with the degree of conversion of monomers into polymers (DC). Fourier transform infrared spectroscopy (FTIR), nanoindentation and atomic force microscopy are usually used to assess the DC [18,19]. Alternatively, the cure effectiveness can be indirectly inferred by the mechanical test named as depth of cure, which was used in this study [20,21,22].

## 2. Materials and Methods

### 2.1. Resin Composites and Photoactivation Techniques

Three models/brands of resin composites were selected for this study. Details regarding each resin composite are shown in Table 1. For simplification, these resins are referred to in this study as Z-250, Charisma and Ultrafill. 

**Figure 1:**
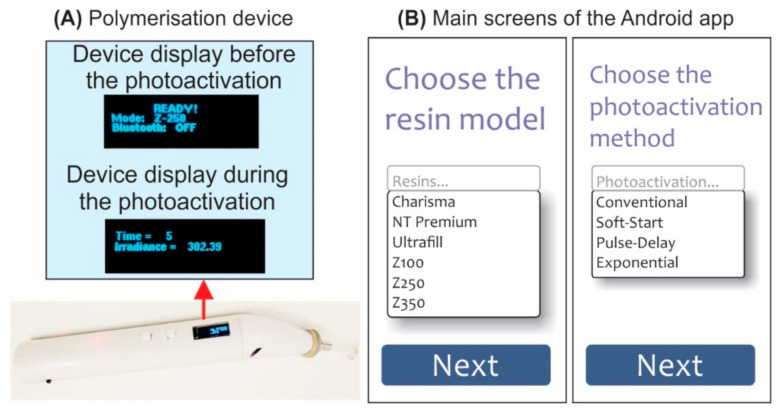
(**A**) Polymerisation device used in this study. (**B**) Main screens of the Android app used in this study (this app is available for free download at the Google Play Store with the name MDev).

Photoactivation processes were performed using a polymerisation device developed by our research group (Patent Registration Number BR1020160078245, INPI, Brazil). This device is equipped with a dental blue LED (LZ4-40B208-0000, LED Engin Inc., San Jose, CA, USA). The curing device was connected to a smartphone via Bluetooth connection. An Android app also developed by our research group (Mdev-System for photopolymerization of dental resin composites, Registration Number BR5120200009891, INPI, Brazil) was used to send the photoactivation parameters to the device, which allowed the control of the LED irradiance during the photoactivation process according to a mathematical function, or the use of the classical photoactivation protocols. Output irradiance was gauged using a radiometer (RD7, Ecel Indústria e Comércio Ltd.a; Ribeirão Preto, São Paulo, Brazil). Figure 1 shows an image of the polymerisation device and the main screens of the Android app used in this study.

The total photoactivation energy (*E_TOTAL_*) was obtained from the resin manufacturer’s recommendations. The other photoactivation parameters (as time of pulse and time of delay) were determined for each photoactivation technique (conventional, soft-start and pulse-delay) from the consensus of the literature, which showed that irradiances generally start with half the value of the total irradiance of the photoactivator in the pulse-delay and soft-start protocols. For the conventional, soft-start and pulse-delay protocols, the light-curing device emits constant irradiance as a function of time (or time intervals) and total photoactivation energy given by the product of irradiance and exposure time. The parameters for the exponential photoactivation method, however, were determined using the model of the exponential function proposed by Guimarães et al. [10], which allows determining the instantaneous irradiance during the photoactivation (*i*) as a function of the time (*t*):

(1) $$i\left(t\right)\;=\;a\;\cdot \;e^{\frac{t}{b}}+i_{0},$$

where *a* = 0.58309, *b* = 4.928 and *i*_0_ = 300. The last term in Equation (1) (*i*_0_) corresponds to the irradiance at the start point of the photoactivation process (300 mW/cm^2^). The total time of photoactivation for each technique was determined by simple integration of Equation (1) so that the activation energy reached the expected values, as presented in Equation (2):

(2) $$\int_{0}^{t_{final}}\left(0.58309\;\cdot \;e^{\frac{t}{4.928}}+300\right)dt-E_{TOTAL},$$

**Figure 2:**
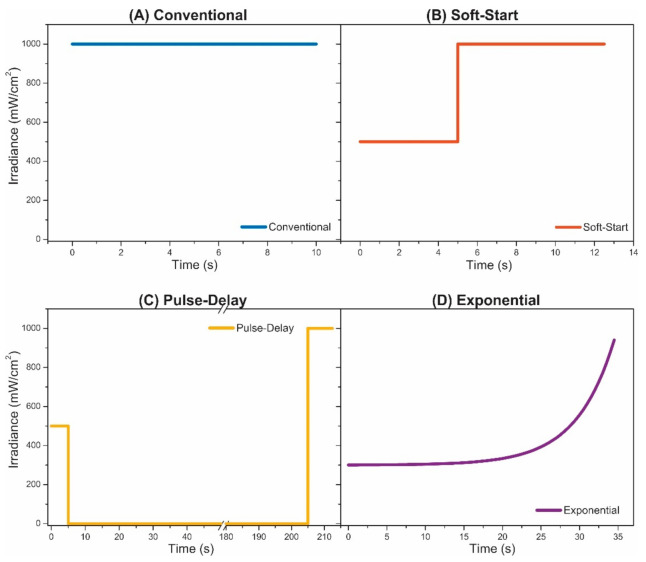
Representation of the irradiance variation as a function of time for the photoactivation protocols: (**A**) conventional, (**B**) soft-start, (**C**) pulse-delay and (**D**) exponential.

In Equation (2), time (*t*) is the only integration variable for adjusting the function to the activation energies. All photoactivation parameters were summarized in Table 2 and the variation pattern of the irradiance over time for each photoactivation protocol is illustrated in Figure 2.

### 2.2. Monitoring of the Polymerisation Shrinkage Stress

Monitoring of the polymerisation shrinkage was performed using an Emic DL 3000 universal testing machine (EMIC Equipamentos e Sistemas de Ensaios Ltd.a, São José dos Pinhais, PR, Brazil). Two steel bases were coupled to the equipment arms and adjusted for insertion and polymerisation of the resin [23]. The gap between the two bases, into which the resin was inserted, was set to 1 mm, meaning that the total volume of resin used in the experiments was 12 mm^3^ (6 mm × 2 mm × 1 mm). The device tip was positioned on the 6 mm side of the steel base. The distance for photopolymerisation was 2 mm.

During the polymerisation process, the controller software traced the curve of the stress force as a function of the polymerisation time. Although photoactivation is usually performed within 50 s, the monitoring time was 300 s, as the resin shrinkage occurs even after activation. The assays were performed in triplicate for each resin model/brand and photoactivation method.

### 2.3. Monitoring of the Resin/Tooth Temperature Variation

Healthy molar human teeth extracted by orthodontists’ indication were used to monitor/evaluate the temperature variation during the resin photoactivation processes. All patients who provided teeth for the present study completed a written consent form. The study protocol was approved by the Ethical Research Committee of the Medical School of Botucatu of São Paulo State University-UNESP. (CAAE: 56085916.0.0000.5411/Report: 1.621.713). Cavities with dimensions similar to the specimens used for the shrinkage experiments (6 mm × 2 mm × 1 mm) were made in each tooth. The cavities’ preparation was performed using water-cooled diamond burs (4138, KG Sorensen; Barueri, São Paulo, Brazil). The resins were packed in the tooth cavity in a single increment, and then photoactivated using one of the photoactivation technique described in this study. The monitoring of the resin’s temperature variation during photoactivation was performed using a non-contact temperature sensor (MLX90614 Infra-Red Thermometer TO-39, Melexis, Ypres, Belgium). A microcontroller was used to acquire real-time temperature from the samples. The temperature sensor was focused on and positioned at a distance of 1.5 cm from the resin surface. The temperature monitoring total time was the same used for the polymerisation shrinkage experiments, i.e., 300 s. The experiments were performed in triplicate.

### 2.4. Depth of Cure Evaluation

Experiments to determine the depth of cure were performed according to the methodology described by Alrahlah et al. [20]. Matrices containing a cavity with dimensions of (15 mm × 4 mm × 2 mm) were manufactured. In each experiment, the resin was inserted into the matrix cavity, and the composites were photoactivated just on one side of the matrix. The photoactivator tip was positioned at a distance of 2 mm from the sample during the photoactivation. All specimens were stored for 24 h at 37 °C, in a dry environment with no light incidence. Subsequently, the Vickers Hardness Number (VHN) was measured at three different points on the specimen surface (0 mm) and three different points for the depths of 2 mm and 4 mm. These measurements were performed using a micro-durometer (Model HM-112, Mitutoyo Corp., Tokyo, Japan) applying a load of 300 g for 15 s. All experiments were performed in triplicate.

### 2.5. Statistical Analyses

One-way analysis of variance (or ANOVA) and Tukey post hoc test were applied to evaluate the effects of the 4 photoactivation protocols (independent variable): 1-conventional, 2-soft-start, 3-pulse-delay, and 4-exponential on the shrinkage stress (dependent variable) during the polymerisation of the 3 different resin composites (1-Z-250, 2-Charisma, and 3-Ultrafill). One-way ANOVA and Tukey test were also used to analyse the significant differences of the shrinkage stress (dependent variable) between different materials (independent variable). In addition, two-way ANOVA was used to analyse the interactions between the variables photoactivation protocol and resin composite, by considering the shrinkage stress as the dependent variable. The 30 maximum values of shrinkage stress for each resin composite and photoactivation protocol were selected to statistically compare the shrinkage stress results. The same procedure was used to statistically compare the results of resin/tooth temperature variation.

Subsequently, one-way ANOVA and Tukey post hoc test were also applied to evaluate the effects of the 4 photoactivation protocols (independent variable) on the hardness of the cured resins in function of the depth on the resin (dependent variable). One-way ANOVA and Tukey test were also used to evaluate the significant differences of the hardness (dependent variable) between different resins (independent variable). Multi-factor ANOVA was used to analyse the interactions between the variables photoactivation protocol, resin composite and depth, by considering the hardness as the dependent variable. These analyses were performed considering all hardness values obtained from the experimental replicates.

All statical analyses were performed using the R software considering a confidence level of 95% (version 4.0.4, R Foundation for Statistical Computing, Vienna, Austria).

## 3. Results

### 3.1. Shrinkage Stress

**Figure 3:**
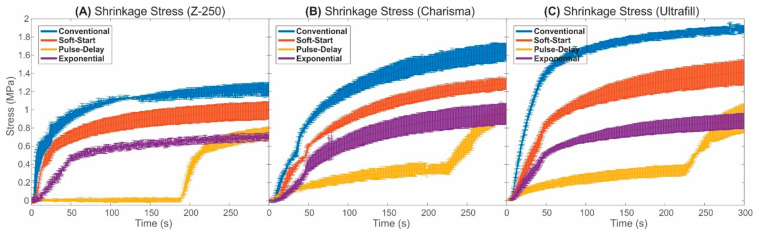
Shrinkage stress along the composite curing process for the resins: (**A**) Z-250; (**B**) Charisma and; (**C**) Ultrafill.

Figure 3 shows a graphical representation of the results obtained from shrinkage stress monitoring for a comparison of the photoactivation techniques used to photopolymerise each brand/model of resin composite evaluated in this study. A summary from one-way ANOVA applied on the shrinkage stress dataset was provided in Table 3. The *p*-value from one-way ANOVA (*p*-value < 0.001) revealed that shrinkage stresses were dependent on the photoactivation protocol applied for the polymerization of all resin composites evaluated in this study. Statistical analyses also showed that the maximum values of shrinkage stress were statistically equivalent for pulse-delay and exponential photoactivation protocols. In addition, the *p*-value from two-way ANOVA showed that the interaction between the variables photoactivation protocol and resin composite was statistically significant (*p* < 0.001).

The results showed consistent patterns in the variation of the shrinkage stress as a function of time for all resin composites. The conventional photoactivation method generated the highest values of stress throughout the monitoring process. The soft-start photoactivation method was able to significantly reduce the stress when compared to the conventional method but was less efficient in minimising the shrinkage stress when compared to the pulse-delay and exponential methods. The comparison of the shrinkage stress obtained by the use of the pulse-delay and exponential methods showed that the stress variation patterns were quite different in the time domain; however, the final and highest values of stress were equivalent for these two photoactivation techniques (see Table 3). 

It should also be noted that the Ultrafill and Charisma resins presented higher values of shrinkage stress when submitted to the conventional photoactivation process. However, when the exponential technique was applied on these composites, the final stresses were comparable with the results obtained for Z-250 resin. In this sense, the variation of the shrinkage stress for the Ultrafill and Charisma were more sensitive to the photoactivation method.

### 3.2. Temperature Variation

**Figure 4:**
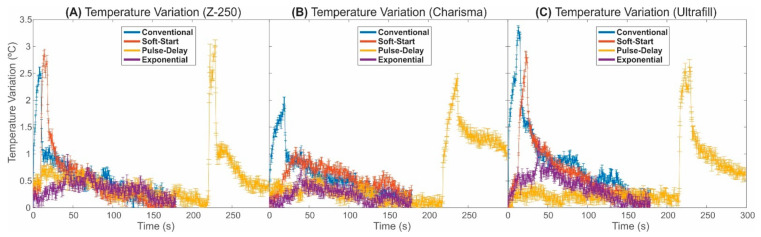
Temperature variation as a function of time on the tooth/resin set for the composites: (**A**) Z-250; (**B**) Charisma and (**C**) Ultrafill.

Figure 4 shows a graphical representation of the temperature variation in the different photoactivation techniques for each brand/model of resin used in this study. one-way ANOVA summary applied on the temperature variation dataset was provided on Table 4. The *p*-value from one-way ANOVA (*p*-value < 0.001) revealed that maximum values of temperature variation were dependent on the photoactivation protocol for all resin composites evaluated in this study. In addition, the *p*-value from two-way ANOVA showed that the interaction between the variables photoactivation protocol and resin composite was statistically significant (*p* < 0.001).

In general, the results showed that the use of the exponential photoactivation method resulted in lower temperature variation when compared to the others photoactivation methods. This result can be rationalised by the photoactivation energy being delivered in a controlled and optimised way in this method. Another relevant observation was that the pulse-delay photoactivation method was not able to minimise the effects of temperature variation (as was observed in the results of shrinkage stress). For this photoactivation technique, the graphs show that the temperature at the final step of the photoactivation process undergoes an increment similar to that observed for the soft-start and pulse-delay methods. Finally, of all the evaluated composites, the photoactivation of the Charisma resin resulted in the lowest increase in temperature during photoactivation (see the statistical comparison in Table 4), despite this resin receiving the highest amount of irradiance energy during photoactivation.

### 3.3. Depth of Cure

**Figure 5:**
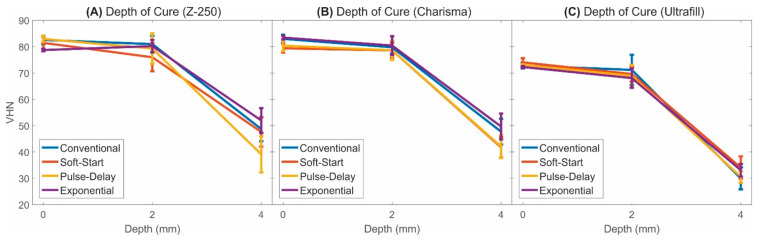
Hardness as a function of depth for the composites after the curing process: (**A**) Z-250; (**B**) Charisma and (**C**) Ultrafill.

VHN as a function of the depth was used to indirectly evaluate the effectivity of the polymerisation, in the way to make inferences about the photoactivation process and to compare the exponential photoactivation technique with the conventional, soft-start, and pulse-delay methods. Figure 5 presents a graphical representation of the results of Vickers microhardness (including standard deviation bars) as a function of specimen’s depth (0 mm, 2 mm, and 4 mm) after the curing process.

One-way ANOVA summary (Table 5) showed there were no statistically significant differences between the hardness at 0 mm and 2 mm for all photoactivation protocols when the resins Z-250 and Charisma were evaluated in isolation. For the Ultrafill resin, there was a statistically significant difference in hardness at the depths of 0 mm and 2 mm, however, this difference was observed for all photoactivation protocols. Additionally, it was observed a significant decrease in hardness for all resin composites at a depth of 4 mm. All these results revealed that the hardness as a function of the depth did not depend on the photoactivation technique (*p*-value > 0.05) but depends on depth (*p*-value < 0.001) for all brands/models of resins evaluated in this study. The *p*-value from multi-factor ANOVA showed that the interaction between the variables photoactivation protocol, resin composite and depth was statistically significant (*p* < 0.001). The results in Table 5 also showed that the hardness at 4 mm for all resin composites and all photoactivation protocols were significantly less than 80% of the respective surface hardness (0 mm), which indicates insufficient polymerisation.

## 4. Discussion

Temperature variation results showed that there was an abrupt increase in temperature during the photoactivation process using the conventional, soft-start, and pulse-delay techniques, and it occurred precisely at the moment that the light irradiance undergoes the maximum values. This result reveals that the resin composites have a low thermal capacity, as the temperature variations are almost instantaneous following the irradiance variation. Although the results showed that the photoactivation process causes an increase in temperature on the set tooth/resin, the highest temperature variation value among all resins observed in this study was approximately 3.5 °C (obtained for the resin Ultrafill, photoactivated by the use of the conventional technique). This increment in the temperature is not enough to cause damage in the restoration area. Studies have shown that damage can be caused when the temperature in the tooth/resin exceeds a variation of 6 °C [14,24]. Thus, it can be concluded that if the activation energy recommended by the manufacturer is used, the temperature variation for any photoactivation method will not cause a problem for the patient when using one of the three resins evaluated in this study.

However, the results of stress revealed that the polymerisation shrinkage can be considered a problem. Studies have shown that shrinkage stress with the same magnitude of the values observed in our results is sufficient to cause adhesion failure at the tooth/resin interface [10,25]. Our results showed that the use of the exponential method significantly reduced the shrinkage stress when compared to using the conventional and soft-start methods. The use of the exponential method was expected to reduce the shrinkage stress when compared to the conventional method, since the conventional technique does not have any type of irradiance control during photoactivation. However, it was observed that the exponential method was more efficient in reducing the shrinkage stress also when compared to the soft-start method, even though the soft-start method is recognized in the literature as a technique with the potential to minimise the shrinkage stress [25,26]. This minimisation of the shrinkage stress can be explained by the low irradiance at the beginning of the photoactivation process, which can extend the polymerisation pre-gel phase and alter the kinetics of the polymerisation chemical reaction [27]. In the pre-gel phase, the molecules are more mobile and can acquire new positions and orientations to compensate the stress generated by the polymerisation shrinkage. In this sense, although the soft-start photoactivation method is like the exponential method (since both techniques use low light at the beginning of the process), there is an abrupt variation in the irradiance during the photoactivation in the soft-start technique, which does not contribute to the minimisation of the shrinkage stress. On the other hand, in the exponential method, the irradiance is practically constant at the beginning of the photoactivation process and is gradually and smoothly increased, being modelled by an exponential curve.

When the exponential method was compared to the pulse-delay method, the obtained results of shrinkage stress were statistically equivalent. However, the two methods differ in their application, because the pulse-delay photoactivation time is excessively long (251 s) and not feasible for practical applications. The exponential method can be performed in less than 1 min, being a favourable time for the routine in dental offices. In addition, if we compare all results from the tests performed using the exponential technique, a standardisation of the shrinkage stress for the different resin brands was observed. This observation can be considered favourable, indicating that the parameters used for the exponential photoactivation technique resulted in a mathematical function close to optimal.

Another relevant result is that the Z-250 composite presented the lowest values for shrinkage stress, even for the conventional photoactivation technique. Composite resin manufacturers have focused on the improvement of dental materials by changing the organic matrices, and by modifying the morphological properties of inorganic charge [28]. For the organic phase, dental composite manufacturers have concentrated on the traditional systems, including Bis-GMA monomers (Charisma), Bis-GMA/TEG-DMA (Z-250) or a TEG-DMA/UEDMA combination (Ultrafill). In addition, viscosity controllers, such as Bis-EMA, PEG-DMA and UDMA are present in the Z-250 product, which can be one of the reasons for the better performance of this composite in the shrinkage stress experiments. In order to minimise shrinkage upon polymerisation and improve wear resistance, several other approaches have been suggested to modify the organic matrix, as well as the filler particle morphology (shape), size range and volume content [29]. Thus, the development of resin composite generations has promoted a classification according to the filler particle size, including macrofill, microfill and hybrid materials [30]. The hybrid resin composites have an average particle size slightly greater than 1 µm, adding a portion of the 40 nm fillers (Charisma and Ultrafill, Table 1). Nanofilled and nanohybrid resins are considered to be a combination of microhybrid and nanofilled sized particles, firstly marketed as the Z-250 range of restorative materials (Table 1). The literature has proposed that finer particle-based materials should provide less inter-particle space, resulting in a relatively smooth wear surface and, likewise, more longevity of material [31,32]. 

An evaluation of the polymerisation efficiency could be performed by analysing the composite hardness at different depths, as the depth of cure is considered a physical property associated with the polymerisation effectiveness [20]. The depth of cure results showed all photoactivation protocols resulted in resins cured with equivalent hardness as a function of the depth. Thus, despite the differences in the mode of energy delivery during the different types of photopolymerisation processes, the curing efficiency was not affected by these parameters. Therefore, the results reported here indicated that there is no harm to the curing process by the choice of an alternative photoactivation method, in cases where the total activation energy is equal to that recommended by the composite manufacturers. In this way, the use of the exponential method of photoactivation (which presented better results in the shrinkage stress and temperature variation experiments) can be considered as a viable alternative for practical use. However, it is worth mentioning that there was a significant reduction of the hardness values for all specimens to a depth of 4 mm. This occurred because the resin layers deeper under the top surface receive progressively lower amount of photons that will activate the photoinitiator molecules [23]. When the surface is polymerized, the patterns of the light absorption and dispersion are changed, which results in a low conversion of the monomers into polymers in the bulk of the resin [6,33,34]. It should be noted that the hardness at 4 mm for all resin composites and all photoactivation protocols were significantly less than 80% of the respective surface hardness (0 mm), which was previously interpreted as insufficient polymerisation [20,35]. Insufficient polymerisation or curing may result in poor bonding with the hard dental tissue and microleakage [6,21]. Indirectly, it can be inferred from these results that, if the dental cavity is deep, the restorations applying the activation energies recommended by the resins manufacturers must be performed in increments [36].

All these results can be used to subsidise the standardisation of the photoactivation process. Reported studies have proposed photoactivation processes using parameters with large variances, particularly for activation energy (with values ranging between 10–60 J) [22,37,38,39,40,41,42], and the high activation energies can overcure the resin and maximise shrinkage. Therefore, it can be observed that there are not standard protocols for photoactivation, and none of the referred-to studies present a systematic method with which to determine the optimal photoactivation parameters. In general, photoactivation parameters are proposed without a clear criterion. In this sense, in our study, we do not propose changes in the total activation energy, but in the way in which this energy is delivered to the composite surface. Our results have shown that the optimal photopolymerisation process was achieved when the exponential photoactivation protocol was applied, using the total energy recommended by the composite manufacturer.

On the other hand, this study had some limitations. Studies have reported that storage conditions of the specimens after the photoactivation can affect their mechanical properties and conversion degree [43,44,45]. Other important mechanical and physico-chemical properties of the resins after the curing process were not evaluated in this study, such as flexural strength, surface roughness, degree of conversion, nanohardness (which may be more informative than Vickers microhardness, since this technique allow the evaluation of hardness at the nanoscale), and residual monomers [6,46,47,48]. These are some limitations that can be addressed in future studies.

## 5. Conclusions

The results obtained in this study demonstrated that controlling the irradiance during the photoactivation process has a direct influence on the dynamic behaviour of the shrinkage stress and temperature variation during the resin composite polymerisation. The use of exponential and pulse-delay photoactivation techniques promoted better results than the use of conventional and soft-start methods and favoured a significant reduction in the polymerisation shrinkage stress. However, the pulse-delay method requires a relatively long photoactivation time, which impairs its application and adoption by professionals in the dental restoration area.

The increments in the temperature were lower when the exponential photoactivation was applied. However, the temperature variation in the tooth/resin set during the photoactivation process were not sufficient to cause damage in the region of restoration for all photoactivation protocols. Thus, it can be concluded that if the activation energy recommended by the manufacturer is used, the temperature variation is not characterised as a problem for the patient when using one of the three resins evaluated in this study.

The evaluation of the polymerisation efficiency by analysing the composite surface hardness showed that all photoactivation protocols resulted in resins cured with equivalent hardness, indicating that the choice of alternative protocol will not harm the polymerisation.

All the results indicate that the exponential photoactivation method is the best alternative for resin composite photoactivation processes. This method can be adjusted for each type/brand of resin composite, promoting an operational standardisation of the photoactivation process, which has not been previously reported.

## Figures and Tables

**Table 1:** Characteristics of each resin composite used in this study.

Resin	Manufacturer	Classification	Organic Matrix ^1^	Inorganic Charge
Filtek Z-250 XT	(3M ESPE, St. Paul, MN, USA)	Nanohybrid	Bis-GMA TEG-DMA Bis-EMA PEG-DMA UDMA	Surface-modified Zirconia/silica (0.01–3.0 µm) Non-agglomerated/non-aggregated SiO_2_ (0.02 µm) Filler loading (68% volume)
Charisma Classic	(Heraeus Kulzer, Hanau, Germany)	Microhybrid	Bis-GMA	Microglass^®^ Barium aluminium fluoride glass Particle size (0.005–10 µm) Filler loading (61% volume)
Ultrafill	(Biodinâmica Ltd.a, PR, Brazil)	Microhybrid	TEG-DMA UDMA	Inorganic charge (0.04–2.2 µm) Filler loading (79% volume)

^1^ Bis-EMA, ethoxylated bisphenol A glycol dimethacrylate; Bis-GMA, bisphenol A glycidyl dimethacrylate; PEG-DMA, polyethylene glycol-dimethacrylate; TEG-DMA, triethylene glycol dimethacrylate; UDMA, urethane dimethacrylate; UEDMA, urethane ethyl dimethacrylate.

**Table 2:** Photoactivation parameters for all techniques/resins in our study. The variable *i* corresponds to irradiance.

Resin	*E_TOTAL_* (J)	Conventional	Soft-Start	Pulse-Delay	Exponential
Z-250	10	Step 1: *i*: 1000 mW/cm^2^ Time: 10 s	Step 1: *i*: 500 mW/cm^2^ Time: 5 s Step 2: *i*: 1000 mW/cm^2^ Time: 7.5 s	Step 1: *i*: 500 mW/cm^2^ Time: 5 s Step 2: *i*: 0 mW/cm^2^ Time: 200 s Step 3: *i*: 1000 mW/cm^2^ Time: 7.5 s	Step 1: *i*: defined by Equation (2) Time: 30 s
Charisma	20	Step 1: *i*: 1000 mW/cm^2^ Time: 10 s	Step 1: *i*: 500 mW/cm^2^ Time: 10 s Step 2: *i*: 1000 mW/cm^2^ Time: 15 s	Step 1: *i*: 500 mW/cm^2^ Time: 10 s Step 2: *i*: 0 mW/cm^2^ Time: 200 s Step 3: *i*: 1000 mW/cm^2^ Time: 15 s	Step 1: *i*: defined by Equation (2) Time: 39 s
Ultrafill	16	Step 1: *i*: 1000 mW/cm^2^ Time: 16 s	Step 1: *i*: 500 mW/cm^2^ Time: 10 s Step 2: *i*: 1000 mW/cm^2^ Time: 11 s	Step 1: *i*: 500 mW/cm^2^ Time: 10 s Step 2: *i*: 0 mW/cm^2^ Time: 200 s Step 3: *i*: 1000 mW/cm^2^ Time: 11 s	Step 1: *i*: defined by Equation (2) Time: 37 s

**Table 3:** Mean and standard deviation of 30 maximum values of shrinkage stress. Different letters indicate statistically significant differences (*p*-value < 0.05). Uppercase letters compare the effect of photoactivation protocol on the shrinkage stress (within lines), and lowercase letters compare shrinkage stress for each resin composite (within columns).

Resin	Conventional (Mean ± SD) MPa	Soft-Start (Mean ± SD) MPa	Pulse-Delay (Mean ± SD) MPa	Exponential (Mean ± SD) MPa
Z250	1.24 ± 0.05 ^A,a^	0.99 ± 0.08 ^B,a^	0.74 ± 0.06 ^C,a^	0.73 ± 0.02 ^C,a^
Charisma	1.64 ± 0.08 ^A,b^	1.29 ± 0.05 ^B,b^	0.96 ± 0.07 ^C,b^	0.96 ± 0.10 ^C,b^
Ultrafill	1.91 ± 0.03 ^A,b^	1.41 ± 0.11 ^B,b^	0.90 ± 0.13 ^C,b^	0.88 ± 0.07 ^C,b^

**Table 4:** Mean and standard deviation of 30 maximum values of temperature variation. Different letters indicate statistically significant differences (*p*-value < 0.05). Uppercase letters compare the effect of each photoactivation protocol on the temperature variation (within lines), and lowercase letters compare the temperature variation for each resin composite (within columns).

Resin	Conventional (Mean ± SD) °C	Soft-Start (Mean ± SD) °C	Pulse-Delay (Mean ± SD) °C	Exponential (Mean ± SD) °C
Z250	2.39 ± 0.11 ^A,a^	2.72 ± 0.11 ^B,a^	2.80 ± 0.21 ^B,a^	0.75 ± 0.09 ^C,a^
Charisma	1.84 ± 0.10 ^A,b^	1.01 ± 0.05 ^B,b^	2.23 ± 0.09 ^C,b^	0.59 ± 0.08 ^D,b^
Ultrafill	3.14 ± 0.18 ^A,a^	2.61 ± 0.19 ^B,a^	2.49 ± 0.09 ^B,a^	0.98 ± 0.15 ^C,a^

**Table 5:** Mean and standard deviation of resin hardness as a function of the depth. Different letters indicate statistically significant differences (*p* < 0.05). Uppercase letters compare the effect of each photoactivation protocol on the hardness (within lines), and lowercase letters compare the effect of the resin composite on the hardness (within columns). * indicate relative hardness statistically <80% of the hardness at 0 mm.

Resin	Depth	Conventional (Mean ± SD) HV	Soft-Start (Mean ± SD) HV	Pulse-Delay (Mean ± SD) HV	Exponential (Mean ± SD) HV
Z250	0 mm	82.5 ± 1.9 ^A,a^	81.4 ± 2.7 ^A,a^	82.9 ± 1.4 ^A,a^	81.8 ± 0.8 ^A,a^
Charisma	0 mm	83.0 ± 1.8 ^A,a^	79.4 ± 1.9 ^A,a^	80.4 ± 2.4 ^A,a^	83.4 ± 0.9 ^A,a^
Ultrafill	0 mm	72.8 ± 1.3 ^A,b^	74.0 ± 1.8 ^A,b^	73.1 ± 1.3 ^A,b^	72.3 ± 0.7 ^A,b^
Z250	2 mm	81.0 ± 3.6 ^A,a^	76.0 ± 6.1 ^A,a^	79.3 ± 6.7 ^A,a^	80.2 ± 2.8 ^A,a^
Charisma	2 mm	79.8 ± 2.8 ^A,a^	78.6 ± 3.2 ^A,a^	78.6 ± 4.2 ^A,a^	80.4 ± 4.1 ^A,a^
Ultrafill	2 mm	71.2 ± 6.7 ^A,b^	69.6 ± 3.4 ^A,b^	68.9 ± 4.8 ^A,b^	68.1 ± 4.3 ^A,b^
Z250	4 mm	48.7 ± 5.3 ^B,a,^*	47.6 ± 6.4 ^B,a,^*	39.1 ± 7.9 ^B,a,^*	52.0 ± 5.4 ^B,a,^*
Charisma	4 mm	47.7 ± 5.6 ^B,a,^*	41.8 ± 4.5 ^B,a,^*	42.1 ± 5.1 ^B,a,^*	49.7 ± 5.7 ^B,a,^*
Ultrafill	4 mm	30.0 ± 4.8 ^B,b,^*	34.1 ± 4.9 ^B,b,^*	30.5 ± 2.5 ^B,b,^*	33.1 ± 2.8 ^B,b,^*
